# Focal Intramucosal Adenocarcinoma Occurring in Gastric Hyperplastic Polyps: Two Case Reports

**DOI:** 10.1155/2015/201042

**Published:** 2015-08-04

**Authors:** Keisuke Taniuchi, Mitsuo Okada, Hiroshi Sakaeda

**Affiliations:** ^1^Department of Gastroenterology, Chikamori Hospital, Kochi 780-8522, Japan; ^2^Department of Endoscopic Diagnostics and Therapeutics, Kochi Medical School, Kochi University, Nankoku 783-8505, Japan

## Abstract

Gastric hyperplastic polyps are generally considered benign lesions, although rare cases of adenocarcinoma have been reported. Two cases of intramucosal adenocarcinoma originating from gastric hyperplastic polyps that were successfully removed by endoscopic mucosal resection or endoscopic submucosal dissection are reported. On pathological examination, adenocarcinoma limited to the hyperplastic foveolar epithelial mucosa of the gastric hyperplastic polyps was observed.

## 1. Introduction

Hyperplastic polyps (HPs) are the most common type of polypoid lesion of the stomach. Until recently, gastric HPs were considered to be insignificant in terms of potential malignant transformation, and the incidence of malignant change has been reported to be relatively low, with an average of only 2.1% in large series [1]. There have been increasing numbers of reports of dysplasia and carcinoma arising within HPs, with rates of occurrence varying from 1.5% to 4.5% [2]. Gastric HPs larger than 1 cm are said to have an increased risk of malignant transformation [3]. Therefore, HPs are sufficiently common to warrant careful attention because of their association with dysplasia and gastric cancer.

HPs are inflammatory proliferations of gastric foveolar cells. The classic association of gastric HPs has been with mucosal atrophy, whether caused by* Helicobacter pylori* (*H. pylori*) infection or autoimmune gastritis [4]. In recent years, the proportion of gastric HPs occurring in the setting of normal or reactive gastric mucosa with no evidence of current or prior* H. pylori* infection has been increasing [5]. Carcinomas arising in relation to gastric HPs are usually well differentiated, although some cases of poorly differentiated and signet-ring carcinomas have been reported [6].

Two cases of gastric HP with intramucosal adenocarcinoma are presented. In these cases, the neoplastic transformation occurred in the hyperplastic foveolar epithelium of the gastric HPs.

## 2. Case Report

**Figure 1:**
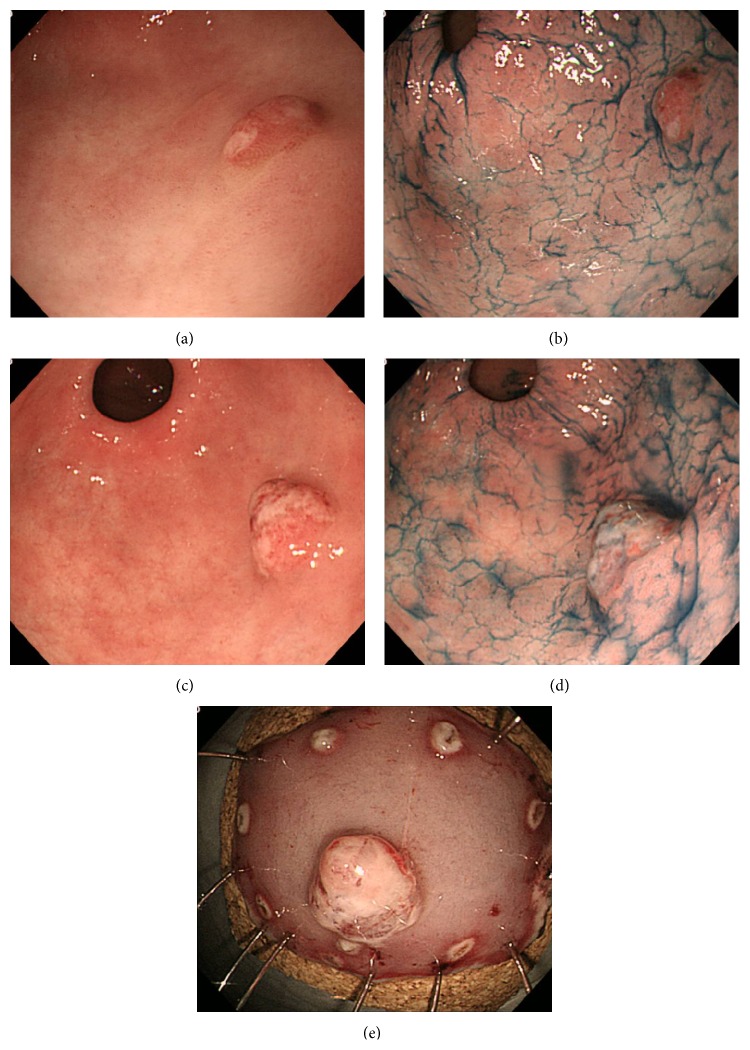
Case 1, endoscopic examination. EGD examination in April 2013 ((a) white-light endoscopy; (b) white-light endoscopy with indigo-carmine dye staining). EGD examination in April 2014, when an initial diagnosis of focal cancer within gastric HP was made ((c) white-light endoscopy; (d) white-light endoscopy with indigo-carmine dye staining). Endoscopic findings after* en bloc* resection by ESD (e). EGD: esophagogastroduodenoscopy; HP: hyperplastic polyp; ESD: endoscopic submucosal dissection.

**Figure 2:**
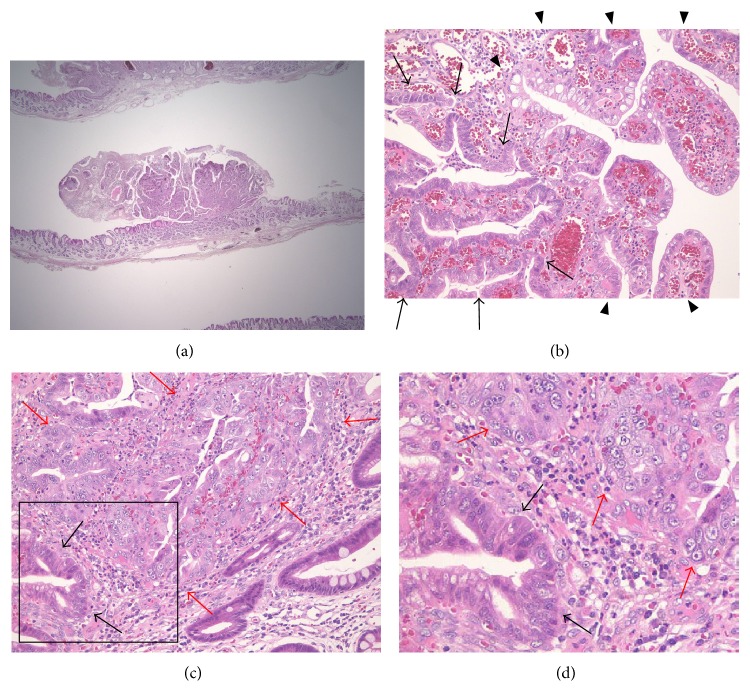
Case 1, histopathological findings. (a) Resected specimen obtained by endoscopic ESD (H&E, ×4). (b) Foci of tubular adenoma with mild dysplasia (between the arrows) in the setting of HP (between arrowheads) are noted (H&E, ×20). (c) Foci of carcinomatous transformation (between red arrows) adjacent to the adenomatous lesion (black arrows) in sequence are observed (H&E, ×20). (d) The black box in (c) depicts the position of the enlarged section. Red arrows: foci of carcinomatous transformation; black arrows: adenomatous lesion. ESD: endoscopic submucosal dissection; HP: hyperplastic polyp.

*Case 1*. A 73-year-old woman was found to have a gastric HP on the posterior wall of the gastric antrum during a screening esophagogastroduodenoscopy (EGD) performed in April 2010. She had no symptoms, and her family history was unremarkable. The HP was 15 mm in size and classified as type II (Yamada classification). Thereafter, she underwent annual EGDs. The EGD performed 3 years later revealed that the gastric HP remained the same size (Figures 1(a) and 1(b)); a biopsy revealed mild dysplasia in the hyperplastic foveolar epithelium of the gastric HP. The following EGD, performed 4 years later, revealed a significant increase in size, to 20 mm, and the lesion was classified as type III (Yamada classification) (Figures 1(c) and 1(d)). At this time, a biopsy revealed focal adenocarcinoma in the HP. Routine laboratory examination results were within normal limits, and the serum carcinoembryonic antigen (CEA) level was not elevated. The polyp was resected* en bloc* by endoscopic submucosal dissection (ESD) performed with a hook knife (Figure 1(e)). The resected specimen revealed well-differentiated adenocarcinoma limited to the mucosa around the elongated, grossly distorted, branching, and dilated hyperplastic foveolae lying in an edematous stroma rich in vasculature and small, haphazardly distributed, smooth muscle bundles with dysplastic foci (Figures 2(a)–2(d)). The lesion was 20 mm in maximum diameter, and it was completely removed with an excision margin greater than 2 mm that was free of tumor cells. The polyp with focal adenocarcinoma was classified as early stomach cancer, pT1a(M), Tub1, ly0, v0, pN0, pM0, and pStage IA, in accordance with the Japanese Classification of Gastric Carcinoma (JCGC). Histopathological assessment of biopsy specimens collected from the mucous membrane of the antrum and body of the stomach showed chronic gastritis with no* H. pylori* infection. Serum anti-*H. pylori* antibody was less than 3 U/mL.

**Figure 3:**
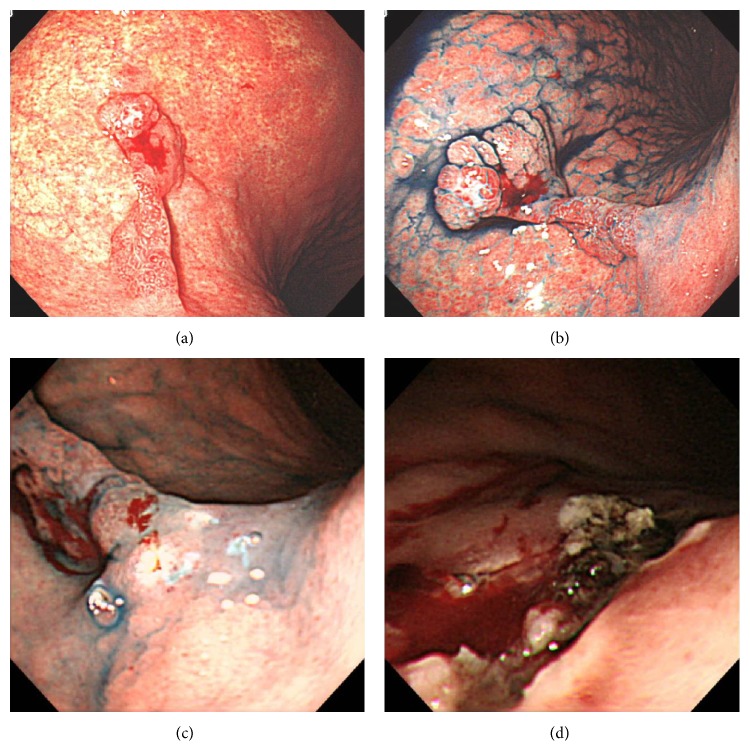
Case 2, endoscopic examination. EGD examination in October 2014, when an initial diagnosis of focal cancer within gastric HP was made ((a) white-light endoscopy; (b) white-light endoscopy with indigo-carmine dye staining). Endoscopic findings after* en bloc* resection by EMR (c, d). EGD: esophagogastroduodenoscopy; HP: hyperplastic polyp; EMR: endoscopic mucosal resection.

**Figure 4:**
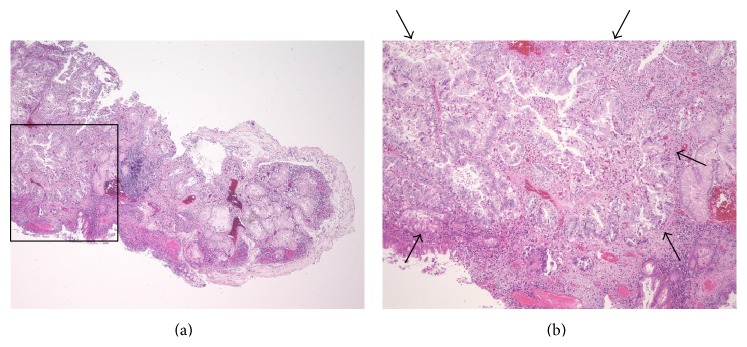
Case 2, histopathological findings. (a) Resected specimen obtained by endoscopic EMR (H&E, ×4). (b) The black box in (a) indicates the region shown in the enlarged image. Foci of carcinomatous transformation (between arrows) in the setting of HP are observed (H&E, ×20). EMR: endoscopic mucosal resection; HP: hyperplastic polyp.

*Case 2*. A 66-year-old woman was found to have a gastric HP on the posterior wall of the proximal gastric body during a screening EGD performed in October 2011. She had no symptoms. Her family history was unremarkable, and she was a housekeeper. The HP was classified as type IV (Yamada classification). Thereafter, she underwent annual EGDs. The EGD performed 3 years later (Figures 3(a) and 3(b)) revealed the gastric HP to be unchanged in size, 20 mm, compared to the EGD performed 2 years later, when a biopsy had revealed no dysplasia or cancer cells. However, a biopsy taken at this time revealed focal adenocarcinoma in the HP. Routine laboratory examination results were within normal limits, and the serum CEA level was not elevated. The polyp was resected by endoscopic mucosal resection (EMR) using a submucosal saline injection technique for reduction of iatrogenic thermal injury, and, to increase the probability of a truly curative complete resection, the polyp was lifted up and removed* en bloc* with a diathermic loop (Figures 3(c) and 3(d)). The lesion was 20 mm in maximum diameter, and the resection margin was free of tumor cells. The resected specimen revealed focal well-differentiated adenocarcinoma limited to the mucosa around the hyperplastic foveolae lying in a vascular edematous stroma (Figures 4(a) and 4(b)). No dysplasia, intestinal metaplasia, or* H. pylori* infection was identified in the benign gastric epithelium of the polyp. The lesion was classified as early stomach cancer, pT1a(M), Tub1, ly0, v0, pN0, pM0, and pStage IA, according to the JCGC. Histopathological assessment of biopsy specimens collected from the mucous membrane of the antrum and the body of the stomach showed chronic gastritis with no* H. pylori* infection. Serum anti-*H. pylori* antibody was less than 3 U/mL.

In the present cases, the HPs were removed completely by ESD or EMR and the patients did not require any additional surgery because the tumors were limited to the mucosal layer.

## 3. Discussion

Two cases of rare focal adenocarcinoma arising in gastric HPs were presented. There have recently been increasing numbers of reports of dysplasia and carcinoma arising within gastric HPs. During the malignant transformation of gastric HPs, cancer may spontaneously occur in the lesion through multistep carcinogenesis, such as via the hyperplasia-adenoma (dysplasia)-adenocarcinoma sequence [7]. Chromosomal aberrations, microsatellite instability, and p53 mutations were found to be mutually exclusive in adenomatous areas arising in gastric HPs; hence, there has been speculation regarding whether these could be alternate pathways of gastric carcinogenesis [1, 8, 9]. However, whether gastric HP carcinoma usually arises from a precancerous lesion or* de novo* remains unknown [2]. Current Case 1 had focal dysplasia situated close to the focal adenocarcinoma, which appears to confirm the theory of malignant conversion of HPs, that is, hyperplasia-dysplasia-adenocarcinoma. In contrast, current Case 2 had only adenocarcinoma cells without accompanying adenoma or other types of cancer cells. This case supports the existing hypothesis of a sequential progression from a benign HP to cancer. HPs have been reported in association with various types of chronic gastritis, particularly with autoimmune gastritis [10] and in* H. pylori* gastritis [11]; however, both gastric HPs in this report arose from chronic gastritis without intestinal metaplasia or* H. pylori* infection. Therefore, the settings of both cases were similar. The mechanism of carcinogenesis within gastric HPs is uncertain, but the present cases suggest that carcinoma can arise from dysplasia and from hyperplastic foveolar epithelium within gastric HPs.

HPs are usually asymptomatic and are typically found incidentally during routine endoscopic examination [12], as with the current asymptomatic gastric HP cases. Focal adenocarcinomas in these HPs were diagnosed by endoscopic biopsy during careful follow-up observation with EGD. Since attempts to identify molecular changes in gastric HPs have been hampered by the small number of reported cases, a marker for the malignant transformation of these lesions has not yet been found. Therefore, the most important prognostic factor is size: those larger than 1 cm are said to have an increased risk of malignant transformation [9, 13]. Accordingly, the potential association of HP with gastric cancer should generally be taken into consideration, and endoscopists should aggressively obtain biopsy specimens from gastric HPs larger than 1 cm in size. Since the results of biopsy specimen analysis may be falsely negative, all gastric HPs exceeding 2 cm (or, according to some opinions, even 5 mm) in size should always be resected [14]. Other authors recommend the resection of gastric HPs that are larger than 1 cm in size to obtain an accurate diagnosis [15, 16].

The current cases illustrate that gastric HP may be associated with gastric cancer and that the biopsy-first approach is reasonable, because it allows definitive diagnosis and planning of treatment according to pathology results after consultation with the patient. The decision of whether endoscopic or surgical resection is needed must be made, and the endoscopist should consider careful follow-up and/or complete removal of all HPs that measure 1 cm or more.
